# The effects of CPET-guided cardiac rehabilitation on exercise tolerance in older persons with CHD after PCI

**DOI:** 10.1038/s41598-023-47494-x

**Published:** 2023-11-15

**Authors:** Tao Shen, Yuwei Wang, Chuan Ren, Yanxin Song, Wei Gao, Fen Liu, Gang Li, Zhimin Wei, Peng Wang, Wei Zhao

**Affiliations:** 1 Department of Cardiology, Peking University Third Hospital, NHC Key Laboratory of Cardiovascular Molecular Biology and Regulatory Peptides, Key Laboratory of Molecular Cardiovascular Science, Ministry of Education, Beijing, China; 2 Health Service Department of the Guard Bureau of the Joint Staff Department, Beijing, China; 3 Physical Examination Center, Peking University Third Hospital, Beijing, China

**Keywords:** Cardiology, Diseases

## Abstract

Prescribing appropriate exercise is an important means to improve the safety and efficacy of cardiac rehabilitation. Improper exercise may induce an increased cardiovascular risk in older persons with coronary heart disease. Cardiopulmonary exercise testing (CPET)-guided cardiac rehabilitation could be helpful for providing clinical evidence for cardiac rehabilitation therapy in older persons after percutaneous coronary intervention (PCI). We retrospectively included older persons who underwent PCI and cardiac rehabilitation based on CPET at the Cardiac Rehabilitation Center of Peking University Third Hospital from January 2014 to December 2019. Patients’ baseline and follow-up clinical data were collected. A total of 403 older persons after PCI were included in the study. The mean age was 80.5 ± 4.3. The mean follow-up time was 12 ± 2 months. During the follow-up period, no significant exercise-related adverse events occurred, and the peak oxygen uptake (VO_2_peak) increased compared with baseline (15.5 ± 3.8 ml/min/kg vs. 17.3 ± 4.1 ml/min/kg). Among the 90 patients (22.2%) without exercise habits at baseline who started regular exercise during follow-up, the improvement in VO_2_peak was most significant, at 3.2 ± 0.4 ml/min/kg. Cardiac rehabilitation based on CPET improved exercise habits and exercise tolerance in older persons with coronary heart disease after PCI.

## Introduction

Cardiac rehabilitation, as an important intervention measure, is of great significance for improving the clinical outcomes of patients with coronary heart disease (CHD)^1–3^. For older persons who undergo revascularization, the cardiovascular risk of heart failure, arrhythmia, and sudden death remain. The cardiovascular risk during exercise may increase due to the added effects of age-related physiological changes. Therefore, prescribing appropriate exercise is an important means to improve the safety and efficacy of exercise rehabilitation.

Cardiopulmonary exercise testing (CPET) could provide essential guidance for determining the aerobic exercise intensity for CHD patients. Additional CPET testing is already being used for exercise prescription even for the elderly. However, there has been insufficient research on its influence in older CHD persons. The purpose of this study was to observe the impact of cardiac rehabilitation guidance based on CPET on cardiopulmonary exercise tolerance and exercise adherence in older persons with CHD after percutaneous coronary intervention (PCI) in the context of standardized drug treatment for CHD. This work will be helpful for providing clinical evidence for cardiac rehabilitation therapy in older persons with CHD after PCI.

## Materials and methods

### General information

**Figure 1:**
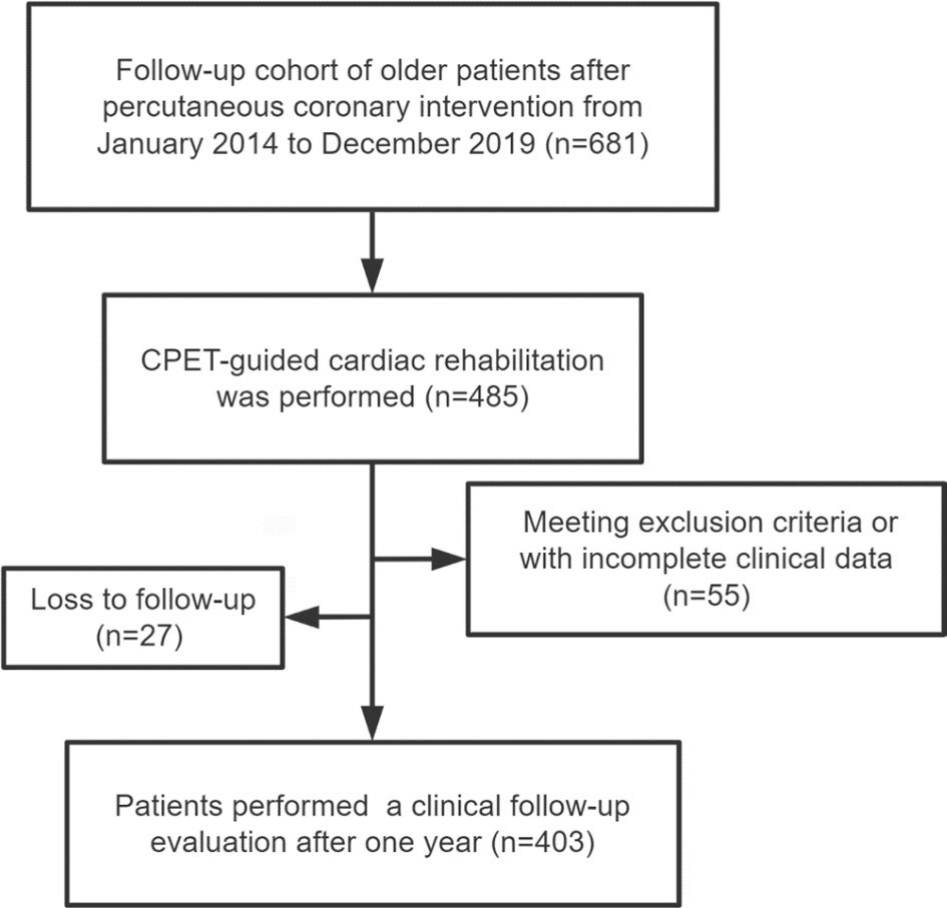
Flowchart of the study.

We retrospectively included CHD patients aged ≥ 75 years who underwent PCI and exercise rehabilitation guided by CPET at the Cardiac Rehabilitation Center of Peking University Third Hospital from January 2014 to December 2019 (Fig. 1).The inclusion criteria were as follows: age ≥ 75 years; successful PCI; CPET-guided cardiac rehabilitation with a clinical follow-up evaluation after one year; and complete clinical data, medication history, biochemical data, and echocardiographic data.

The exclusion criteria were as follows: positive exercise electrocardiogram (ECG); NYHA class III-IV heart function; malignant arrhythmia; valvular heart disease; history of coronary artery bypass graft surgery; and concomitant malignant tumours, haematologic diseases, rheumatic immune diseases, or severe liver or kidney dysfunction.

The study was conducted in accordance with the Declaration of Helsinki. The Ethics Committee of Peking University Third Hospital waived the need for informed consent for the present retrospective study.

A positive exercise ECG was defined as follows: R-wave dominant leads, horizontal or downsloping ST-segment depression ≥ 0.1 mV at 60–80 ms after the J-point, lasting ≥ 2 min; and nonpathological Q-wave leads, upsloping ST-segment elevation ≥ 0.1 mV, lasting ≥ 1 min.

We collected and organized the following patient data: (1) general information, including sex, age, disease diagnosis, height, weight, etc.; (2) past medical history and family history, including history of cardiovascular risk factors such as hypertension, diabetes, hyperlipidaemia, respiratory diseases such as chronic obstructive pulmonary disease, musculoskeletal and neurological disease history, and family history of early-onset CHD, etc.; (3) personal history, including smoking history, exercise habits, etc.; and (4) laboratory test, echocardiography, and CPET results.

### Cardiopulmonary exercise testing

The testing equipment used was the Medgraphics (USA) ULTIMA CardiO2 Cardiopulmonary Exercise System. All patients performed exercise testing using a cycle ergometer, adopting a bicycle exercise protocol. Symptom-limited exercise was encouraged. Patients’ electrocardiogram (ECG), blood pressure, and symptoms were monitored. The entire process was carried out under the supervision of a professional physician.

During the test, continuous recording of patients' ECG, blood pressure, and gas exchange information was performed, including the following parameters: oxygen uptake at anaerobic threshold (VO_2_@AT); heart rate at anaerobic threshold (HR@AT); oxygen pulse at anaerobic threshold; peak oxygen uptake (VO_2_peak); peak heart rate (peak HR); ventilation per carbon dioxide output slope (VE/VCO_2_ slope); oxygen uptake efficiency slope (OUES); and 1-min heart rate recovery.

### Exercise prescription

Individualized exercise rehabilitation prescriptions based on the cardiopulmonary exercise testing system were implemented. Exercise intensity was prescribed using the anaerobic threshold heart rate ± 5 bpm^4^, along with prescriptions for strength training and balance and coordination training. Aerobic exercise modalities such as walking, cycling, and swimming were recommended, with moderate-intensity continuous exercise performed 3–5 days per week for 30–60 min per day, including warm-up, training, and recovery sections.

### Study endpoints

The primary endpoint of this study was the change in patients' exercise tolerance, represented by VO_2_peak and other CPET parameters during the follow-up period. Secondary endpoints included differences in exercise habits. According to the American College of Sports Medicine (ACSM) 10th Edition “ACSM’s Guidelines for Exercise Testing and Prescription”^5^, exercising ≥ 3 times per week, ≥ 30 min per session, and for a duration of ≥ 3 months indicated the presence of exercise habits; those who did not meet this standard were considered to have no exercise habits. Other secondary endpoints included patients' blood test results, echocardiographic parameters, and adverse events during the follow-up period^6^.

### Statistical methods

SPSS 22.0 was used for data analysis and statistics. The Shapiro‒Wilk normality test was performed on all quantitative data in the analysed samples. Normally distributed quantitative data are expressed as the mean ± standard deviation (s), and nonnormally distributed quantitative data are expressed as the median (Q1, Q3). Group comparisons were made using paired t tests and Mann‒Whitney U tests, with paired t tests applied for normally distributed data and Mann‒Whitney U tests for nonnormally distributed data. Categorical variables were analysed using chi-square tests. A two-sided* P* < 0.05 was considered statistically significant.

### Ethical approval

The study was conducted in accordance with the Declaration of Helsinki, and approved by the Ethics Committee of Peking University Third Hospital (Approval Number 2020113).

### Informed consent

Patient consent was waived due to its retrospective nature of observation.

## Results

### General Patient Information

A total of 403 older persons with CHD after PCI were analysed (Table 1). The average age of the patients was 80.5 ± 4.3 years, and there were 305 (75.6%) males. A total of 336 (83.4%) patients had one or more CHD risk factors (including hypertension, diabetes, hyperlipidaemia, and smoking). A total of 134 (16.6%) patients had a history of myocardial infarction. Complete revascularization was achieved in 316 (78.4%) patients. A total of 185 (45.9%) patients were classified as NYHA functional class II.

**Table 1:** Clinical information of older CHD patients after PCI.

Parameters	Total (%), mean ± SD
Age (years)	80.5 ± 4.3
Male, N (%)	305 (75.6)
BMI (Kg/m^2^)	25.1 ± 3.5
History of myocardial infarction, N (%)	134 (16.6)
Complete revascularization, N (%)	316 (78.4)
NYHA class II, N (%)	185 (49.5)
Hypertension, N (%)	271 (67.2)
Diabetes, N (%)	116 (28.7)
Hyperlipidemia, N (%)	262 (65.0)
Smoking history, N (%)	143 (35.4)
Family history of CHD, N (%)	93 (23.0)
Exercise habits, N (%)	256 (63.5)

*CHD* coronary heart disease, *PCI* percutaneous coronary intervention, *BMI* body mass index.

### Comparison of baseline and follow-up patient indicators

General patient data, laboratory indicators, echocardiographic parameters, and CPET indicators were analysed at baseline and during the follow-up period (Table 2). No deaths occurred during the follow-up period. In terms of laboratory indicators, patients had lower low-density lipoprotein (LDL) levels (2.0 ± 0.6 mmol/L vs. 1.4 ± 0.5 mmol/L, *P* = 0.032) and lower N-terminal pro-brain natriuretic peptide (NT-proBNP) levels (305.5 ± 19.4 pg/ml vs. 225.6 ± 11.1 pg/ml, *P* = 0.013) during the follow-up period.

**Table 2:** Characteristics of older persons after PCI during the baseline and follow-up period.

Characteristics	Baseline	Follow-up	*P* value
BMI (kg/m^2^)	25.1 ± 2.9	25.0 ± 2.8	0.726
Laboratory indicators			
Cr (μmol/L)	88.0 ± 11.5	87.4 ± 21.8	0.544
LDL (mmol/L)	2.0 ± 0.6	1.4 ± 0.5	0.032
Hb (g/L)	132.4 ± 16.5	145.1 ± 14.9	0.653
NT-proBNP (pg/ml)	305.5 ± 19.4	225.6 ± 11.1	0.013
Echocardiography indicators			
LVEDD (mm)	47.1 ± 5.6	48.1 ± 3.7	0.565
LVEF (%)	65.7 ± 8.3	66.8 ± 7.6	0.317
Sm (cm/s)	9.3 ± 1.9	9.0 ± 1.5	0.191
E/Em	6.9 ± 2.2	7.5 ± 2.3	0.028
LAP (mmHg)	10.8 ± 2.8	10.1 ± 2.8	0.017
CPET indicators			
VO_2_@AT (ml/min/kg)	12.9 ± 3.7	13.8 ± 4.2	< 0.001
HR@AT (bpm)	98 ± 13	97 ± 11	0.809
VO_2_peak (ml/min/kg)	15.5 ± 3.8	17.3 ± 4.1	0.013
Peak HR (bpm)	118 ± 17	122 ± 16	0.012
Peak SBP (mmHg)	169 ± 24	171 ± 25	0.089
VE/VCO_2_ slope	31.9 ± 5.1	30.9 ± 6.1	< 0.001
OUES	1488.8 ± 395.2	1498.1 ± 328.1	0.453
HRR	17 ± 7	19 ± 8	< 0.001

*PCI* percutaneous coronary intervention, *BMI* body mass index, *Cr* creatinine, *LDL* low density lipoprotein, *Hb* hemoglobin, *NT-proBNP* N-terminal pro-brain natriuretic peptide, *LVEDD* left ventricular end-diastolic dimension, *LVEF* left ventricular ejection fraction, *Sm* systolic velocity of mitral annulus, *E/Em* the ratio of early diastolic transmitral flow velocity to early diastolic tissue velocity, *LAP* left atrium pressure, *VO*_*2*_*@AT* oxygen uptake at anaerobic threshold, *HR@AT* heart rate at anaerobic threshold, *VO*_*2*_*peak* peak oxygen uptake, *Peak HR* peak heart rate, *Peak SBP* peak systolic blood pressure, *VE/VCO*_*2*_* slope* ventilation per carbon dioxide output slope, *OUES* oxygen uptake efficiency slope, *HRR* heart rate recovery.

In terms of echocardiographic parameters, there was no significant change in the left ventricular ejection fraction (LVEF) during the follow-up period (65.7 ± 8.3% vs. 66.8 ± 7.6%, *P* = 0.317), but indicators of left ventricular diastolic dysfunction, i.e., the ratio of early diastolic transmitral flow velocity to early diastolic tissue velocity (6.9 ± 2.2 vs. 7.5 ± 2.3, *P* = 0.028) and left atrium pressure (10.8 ± 2.8 mmHg vs. 10.1 ± 2.8 mmHg, *P* = 0.028), improved.

In terms of cardiopulmonary exercise parameters, patients had higher VO_2_@AT (12.9 ± 3.7 ml/min/kg vs. 13.8 ± 4.2 mmHg, *P* < 0.001), VO_2_peak (15.5 ± 3.8 ml/min/kg vs. 17.3 ± 4.1 mmHg, *P* = 0.013), and peak HR (118 ± 17 bpm vs. 122 ± 16 bpm, *P* = 0.012) values during the follow-up period than at baseline. The VE/VCO_2_ slope, reflecting respiratory efficiency, improved (31.9 ± 5.1 vs. 30.9 ± 6.1, *P* < 0.001), and the 1 min heart rate recovery increased (17 ± 7 bpm vs. 19 ± 8 bpm, *P* < 0.001).

In terms of exercise-related adverse effects, 33 patients (8.3%) had muscle soreness or strain. 17 injurious falls (4.2%) occurred.2 falls (0.05%) resulting in medical care. There were no severe injurious falls or falls resulting in fractures.

Patients were grouped based on whether they had exercise habits during the follow-up period. At the follow-up, patients with exercise habits were younger (80.7 ± 4.3 ml/kg/min vs. 83.9 ± 3.9 ml/kg/min, *P* = 0.007) and had higher ΔVO_2_peak (1.62 ± 0.31 ml/kg/min vs. − 0.02 ± 0.01 ml/kg/min, *P* < 0.001) and VO_2_@AT (14.2 ± 4.1 ml/kg/min vs. 12.9 ± 3.4 ml/kg/min, *P* < 0.001) values than patients without exercise habits. No significant differences were observed between the two groups in terms of laboratory or echocardiographic parameters (Table 3).

**Table 3:** CPET indicators of older persons after PCI during the follow-up period.

Characteristics	With exercise habits (n = 315)	Without exercise habits (n = 88)	*P* value
Age (years)	80.7 ± 4.3	83.9 ± 3.9	0.007
Male (%)	251 (79.6)	54 (61.3)	< 0.001
BMI (kg/m^2^)	25.0 ± 2.9	25.4 ± 3.0	0.736
Laboratory indicators			
Cr (μmol/L)	88.2 ± 13.9	89.1 ± 17.3	0.057
LDL (mmol/L)	1.9 ± 0.6	1.8 ± 0.5	0.609
Hb (g/L)	137.1 ± 15.4	140.3 ± 15.3	0.671
NT-proBNP (pg/ml)	274.7 ± 14.5	251.5 ± 13.8	0.217
Echocardiography indicators			
LVEDD (mm)	47.5 ± 4.9	48.1 ± 4.3	0.513
LVEF (%)	66.0 ± 8.0	67.6 ± 7.9	0.387
Sm (cm/s)	9.2 ± 1.7	8.9 ± 1.7	0.981
E/Em	7.2 ± 2.2	7.8 ± 2.4	0.384
LAP (mmHg)	10.3 ± 2.8	11.1 ± 3.0	0.540
CPET indicators			
VO_2_@AT (ml/min/kg)	14.2 ± 4.1	12.9 ± 3.4	0.002
HR@AT (bpm)	97 ± 12	98 ± 13	0.426
VO_2_peak (ml/min/kg)	17.6 ± 4.0	13.9 ± 4.3	< 0.001
ΔVO_2_peak (ml/min/kg)	1.62 ± 0.31	− 0.02 ± 0.01	< 0.001
Peak HR (bpm)	123 ± 16	119 ± 17	0.026
Peak SBP (mmHg)	170 ± 25	165 ± 27	0.834
VE/VCO_2_ slope	31.2 ± 5.0	31.2 ± 6.7	0.989
OUES	1505.9 ± 350.5	1460.3 ± 362.4	0.289
HRR	20 ± 8	17 ± 9	0.034

*PCI* percutaneous coronary intervention, *BMI* body mass index, *Cr* creatinine, *LDL* low density lipoprotein, *Hb* hemoglobin, *NT-proBNP* N-terminal pro-brain natriuretic peptide, *LVEDD* left ventricular end-diastolic dimension, *LVEF* left ventricular ejection fraction, *Sm* systolic velocity of mitral annulus, *E/Em* the ratio of early diastolic transmitral flow velocity to early diastolic tissue velocity, *LAP* left atrium pressure, *VO*_*2*_*@AT* oxygen uptake at anaerobic threshold, *HR@AT* heart rate at anaerobic threshold, *VO*_*2*_*peak* peak oxygen uptake, *Peak HR* peak heart rate, *Peak SBP* peak systolic blood pressure, *VE/VCO*_*2*_* slope* ventilation per carbon dioxide output slope, *OUES* oxygen uptake efficiency slope, *HRR* heart rate recovery.

### Improvement in patients’ exercise habits

**Figure 2:**
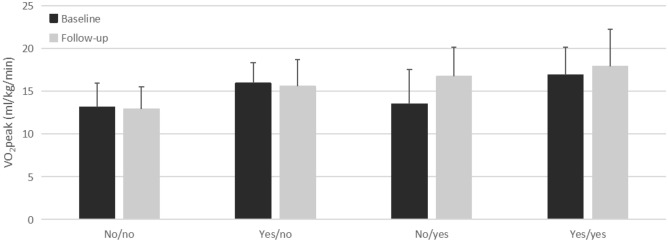
Patients’ exercise habits and VO_2_peak before and after intervention.

Changes in patients’ exercise habits and exercise tolerance from baseline to follow-up were as follows: 57 patients (14.1%) had no exercise habits at baseline or during the follow-up period; 31 patients (7.6%) had exercise habits at baseline but did not maintain them during the follow-up period; 90 patients (22.2%) had no exercise habits at baseline but started regular exercise during the follow-up period; and 225 patients (55.8%) maintained exercise habits at baseline and during the follow-up period. The baseline and follow-up oxygen uptake in each group is shown in Fig. 2, with ΔVO_2_peak values of − 0.2 ± 0.1 ml/kg/min, − 0.3 ± 0.1 ml/kg/min, 3.2 ± 0.4 ml/kg/min, and 1.0 ± 0.2 ml/kg/min, respectively. The improvement in VO_2_peak was most pronounced in patients who had no exercise habits at baseline but developed them during the follow-up period. VO_2_peak did not improve in patients without exercise habits during the follow-up period, regardless of whether they had exercise habits at baseline.

Spearman correlation analysis was performed on patients’ clinical data with ΔVO_2_peak. Exercise habits during the follow-up period (rs = − 0.601,* P* < 0.001), age (rs = − 0.353, P < 0.001), and history of myocardial infarction (rs = − 0.293, *P* = 0.029) were significantly correlated with ΔVO_2_peak, while other clinical data and combined medication use were not significantly correlated with ΔVO_2_peak. Multivariate linear regression analysis was performed on ΔVO_2_peak with the parameters that were significantly correlated with ΔVO_2_peak in the univariate analysis, with the main factors such as exercise habits during the follow-up period as independent variables. The results showed that exercise habits during the follow-up period (B = − 0.406, SE = 0.063, t = − 6.650, *P* = 0.001, *95% CI* − 0.561 to − 0.284), age, and history of myocardial infarction were independent influencing factors of ΔVO_2_peak (Table 4).

**Table 4:** Multiple factor analysis of ΔVO_2_peak.

	*B*	*SE*	*t*	*P*	*95% CI*
Age	− 0.114	0.087	− 6.940	0.023	(− 0.158, − 0.061)
Myocardial infarction history	− 1.054	0.610	− 2.074	0.017	(− 1.391, − 0.772)
With exercise habits during follow-up period	− 0.406	0.063	− 2.650	0.001	(− 0.561, − 0.284)

## Discussion

### Significant clinical benefits of exercise rehabilitation for older persons with CHD

PCI has become the most important means of revascularization for CHD patients, not only effectively improving patients’ clinical symptoms but also significantly reducing the mortality of acute myocardial infarction and high-risk angina patients. However, for older persons, the cardiovascular risk of heart failure, arrhythmia, and sudden death remain after PCI due to the added effects of age-related physiological changes. Compared to non-CHD patients, exercise tolerance is significantly reduced in CHD patients. The decline in exercise tolerance is even more pronounced in older persons with CHD, who may also have mental issues such as depression and dementia, severely affecting their quality of life^7–9^.

Cardiac rehabilitation was an important part of secondary prevention of cardiovascular diseases^10^. There was certain particularity in the implementation of cardiac rehabilitation on different population^11,12^. Previous studies found that increased daily physical activity was associated with a reduced mortality rate in CHD patients, particularly in those who were sedentary, as they experienced greater cardiovascular benefits^13^. Cardiac rehabilitation could promote the physical and mental health of older persons, improving their quality of life^14–17^, and offer benefits for older CHD patients, such as reduced mortality and rehospitalization rates and further revascularization^18,19^. Many studies have reported that aerobic exercise training improves exercise capacity, frailty, and quality of life in older CHD patients^20–23^. However, the patients included in these previous studies were relatively young compared with those in the current study. In particular, in CHD patients aged 75 and older, muscle degeneration and atrophy are more severe; additionally, there is a lack of ligament toughness and elasticity and a greatly reduced capacity for stress responses in the nervous system. Consequently, the risk of exercise-related injuries may increase. Providing an appropriate exercise intensity is a crucial aspect of cardiac rehabilitation, enhancing the efficacy and safety of the treatment.

### Advantages of CPET-guided exercise rehabilitation

CPET is an objective and accurate method for assessing cardiopulmonary function in patients after PCI and is relatively safe^24,25^. For older CHD patients, CPET not only reflects the level of cardiopulmonary function and disease severity but could also be used to assess patients’ balance ability and quality of life^26^. By providing reasonable exercise recommendations based on the collected data, CPET could be used to prescribe exercise rehabilitation programs for older CHD patients, helping to mitigate risk during exercise training.

In the past, the maximum heart rate has often been used as the standard for designing exercise programs in cardiac rehabilitation. It is usually difficult for older persons to achieve their maximum heart rate, and there are unpredictable exercise risks. Moreover, heart rate could be affected by medications such as beta-blockers. A more ideal standard is to individually evaluate patients’ exercise capacity; as exercise capacity improves, CPET could provide guidance for different stages of exercise recommendations.

### Impact of CPET-guided exercise rehabilitation on exercise capacity and exercise habits

In this study, the average age of the patients was 80.5 ± 4.3 years. Exercise intensity was guided based on patients’ anaerobic threshold oxygen uptake during exercise tests, encouraging them to engage in regular, continuous, moderate-intensity exercise. The average follow-up period was 12 ± 2 months, after which patients' exercise habits were reassessed and CPET was repeated. The results showed a significant increase in VO_2_peak and other indicators in the regular exercise group, suggesting that CPET-guided exercise rehabilitation can significantly improve patients’ exercise capacity.

This study found that some patients’ exercise habits changed during the follow-up period after receiving cardiac rehabilitation exercise guidance. At enrolment, 7.6% of patients had exercise habits but did not maintain them during the follow-up period. In contrast, more patients (22%) without exercise habits at enrolment began to develop regular exercise habits during the follow-up period.

In previous research, cardiac rehabilitation increased exercise participation in CHD patients^27^. CPET-based exercise rehabilitation guidance may help older CHD patients gain more confidence in the safety of exercise, thus increasing their enthusiasm for physical activity. Additionally, this study found that patients with exercise habits during the follow-up period had a significantly higher exercise capacity than those without exercise habits. Patients who did not maintain exercise during the follow-up period experienced a decline in exercise capacity due to age or disease progression, which may counteract the benefits gained from their previous regular exercise.

Many medical institutions conduct insufficient exercise assessments and cardiac exercise rehabilitation for older CHD patients due to concerns about exercise-related adverse events or potential risks, which limits the development of cardiac exercise rehabilitation programs. However, this study’s results suggest that even older CHD patients after PCI can safely and effectively improve their exercise capacity through CPET-guided exercise rehabilitation, with the improvement of exercise habits being a crucial aspect. Current research has also explored the use of remote medical devices to improve patients' adherence to cardiac exercise rehabilitation^28,29^. However, due to potential barriers in using remote medical devices and the greater social and economic challenges faced by older persons, further exploration of simpler and more effective methods is needed to enhance the therapeutic effects of exercise rehabilitation in this population.

This study had certain limitations, as it was a single-centre study. The results may have limited generalizability, necessitating further validation in more medical institutions. Additionally, this study was retrospective and did not involve prospective interventional research. The older persons included in the study had few comorbidities and were able to cooperate with cardiopulmonary exercise tests and exercise rehabilitation. As such, the study's conclusions may not apply to older CHD patients with multiple comorbidities, a bedridden status, or a poor exercise capacity. Furthermore, due to a high amount of missing data regarding quality-of-life scores, such as the SF-36 questionnaire scores, these were not included in the statistical analysis.

## Conclusions

Individualized exercise prescription based on CPET is a safe and effective way to guide cardiac rehabilitation in older CHD patients after PCI, improving their exercise capacity and exercise habits.

## Data Availability

The study data are available from the corresponding author upon reasonable request and with the permission of all contributing authors.
